# The progress and future of corneal endothelial transplantation

**DOI:** 10.1007/s10384-024-01083-1

**Published:** 2024-07-31

**Authors:** Toshiki Shimizu, Satoru Yamagami, Takahiko Hayashi

**Affiliations:** Department of Ophthalmology, Department of Visual Sciences, Nihon University School of Medicine, Itabashi, Tokyo Japan

**Keywords:** Corneal endothelial transplantation, Corneal transplantation, DSAEK, DMEK

## Abstract

Endothelial transplantation has recently been accepted worldwide, in the long history of corneal transplantation. The introduction of endothelial keratoplasty (Descemet stripping automated endothelial keratoplasty and Descemet membrane endothelial keratoplasty) has enabled us to expand the surgical indications owing to the low incidence of rejection and quick recovery of visual function. New technologies have been developed to ensure stable postoperative outcomes with a shorter learning curve, such as transplantation using cultured human endothelial cells and induced pluripotent stem cells (iPS) or new devices such as artificial endothelium. This review discusses the history and characteristics of corneal transplantation alongside new treatment options that may offer hope for patients with endothelial disease in the future.

## Introduction

### Corneal anatomy and physiology

**Fig. 1:**
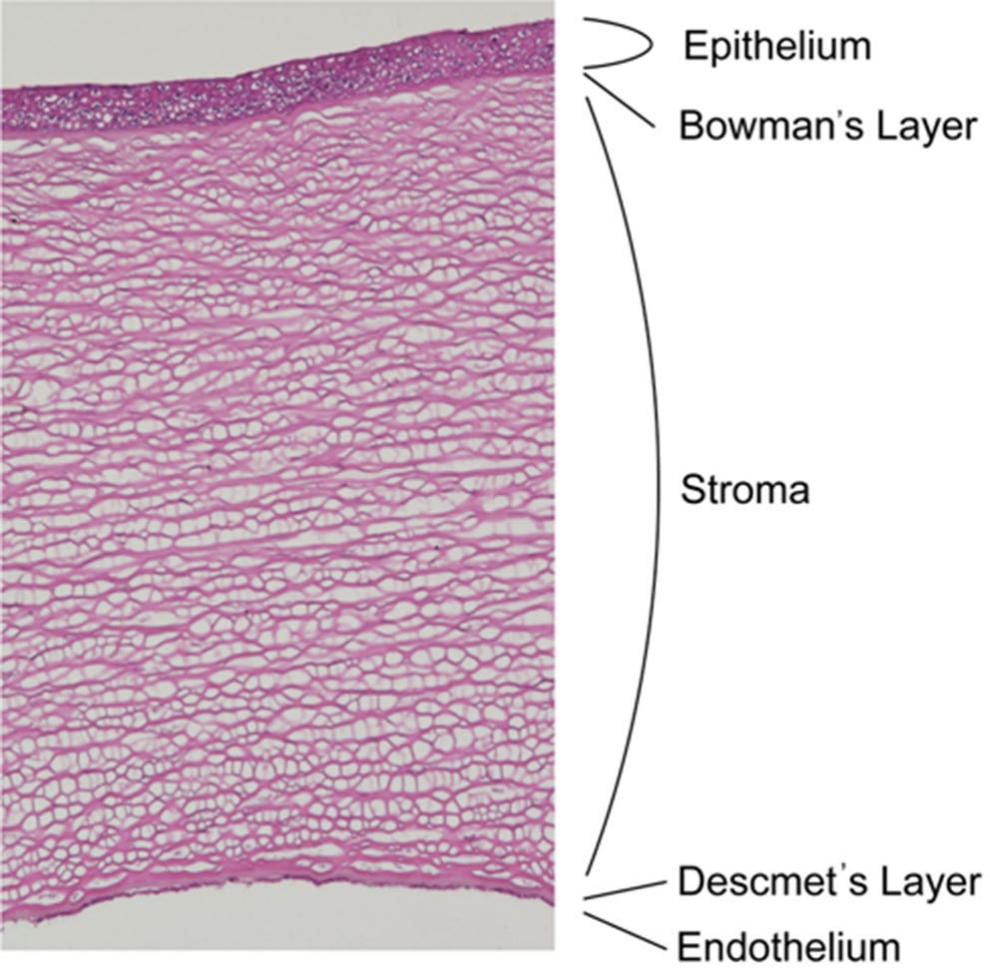
Illustration of corneal structure. From the superficial layer, the epithelium, Bowman layer, stroma, Descemet membrane, and endothelium are arranged in that order. Dendritic cells are resident in the corneal stroma

**Fig. 2:**
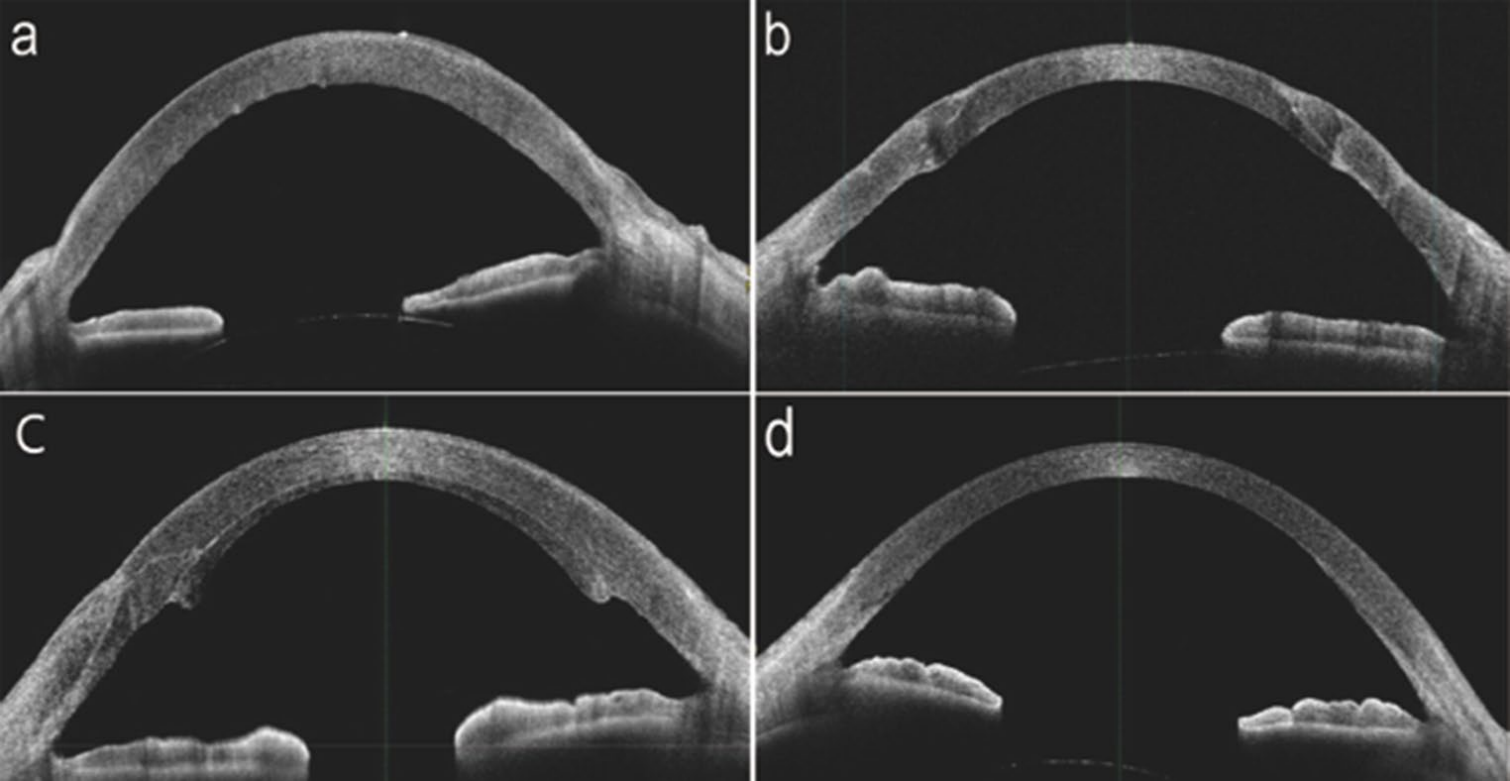
Anterior segment optical coherence tomography (AS-OCT) photographs of each keratoplasty. **a** Bullous keratopathy. The corneal stroma is edematous, and the Descemet membrane is visible on the posterior surface of the cornea. **b** Penetrating keratopathy. The continuity of the cornea is broken. **c, d** Descemet stripping automated endothelial keratoplasty. The graft is adhered to the posterior surface of the cornea, forming a step. Descemet membrane endothelial keratoplasty. The graft is so thin that it is difficult to recognize it with AS-OCT

The cornea is transparent and dome-shaped and, together with the lens, responsible for the refraction of the eye. The cornea consists of 5 layers: the epithelium, Bowman layer, stroma, Descemet membrane (DM), and corneal endothelium (Fig. 1). The corneal epithelium consists of 5 to 6 layers of squamous epithelium with tight junctions between the cells, which mainly act as a barrier against the outside environment. The Bowman layer is a strong membrane with randomly arranged collagen fibers. The corneal stroma, comprising approximately 200 layers of collagen, constitutes more than 90% of the total corneal thickness. In contrast to the Bowman layer, the main components of the corneal stroma are an extracellular matrix of proteoglycans, collagen, and keratocytes. Collagen fibers composed of collagen I and V are regularly arranged to maintain transparency. The DM is a basement membrane secreted by endothelial cells. It is a strong tissue composed mainly of type IV and VIII collagen and is important for maintaining the morphology of the cornea. The corneal endothelium is the innermost layer of flat cells. When observed from the anterior chamber, it is mainly hexagonal in shape. The corneal endothelial cell density (ECD) is approximately 3000 cells/mm^2^ in a healthy cornea at age 20 [1]. When the ECD decreases below 500 cells/mm^2^, corneal transparency cannot be maintained (Fig. 2a).

Endothelial keratoplasty (EK) is required when corneal transparency is reduced owing to endothelial dysfunction, such as in Fuchs endothelial corneal dystrophy (FECD) and bullous keratopathy (BK), or when photophobia and vision loss occur with mild endothelial loss. The development of EK has led to significant advances in corneal transplantation over the past 20 years. In this review, we discuss the history of corneal transplantation and EK, the characteristics of each transplantation procedure, and the future of corneal transplantation.

### History of corneal transplantation

Corneal transplantation is the most common solid organ transplant performed worldwide [2, 3]. The history of penetrating keratoplasty (PKP) is long, with Eduard Zirm performing the first successful all-layer corneal transplantation in 1905 [4]. Subsequently, the number of PKP procedures increased, and it remained the primary option for several decades. Complications specific to PKP, such as postoperative astigmatism, graft rejection, decreased corneal strength, and suture-related infections, can occur and must be properly controlled. Suturing grafts and large wounds represent some of the major postoperative challenges for this procedure.

In diseases with the affected area limited to the corneal endothelium, such as FECD and BK, stromal replacement is unnecessary unless irreversible scarring is observed in the stroma. This concept led to the development of the first corneal endothelial transplant by Tillett in 1956 [5]. In 1998, Melles and colleagues invented a method of pneumatic graft adhesion named posterior lamellar keratoplasty (PLK), in which the posterior corneal lenticle includes the posterior corneal stroma, Descemet membrane, and endothelium [6]. Terry and colleagues introduced PLK for the first time in the United States and called it deep lamellar endothelial keratoplasty (DLEK); in this procedure, the superficial layer of the cornea is removed, and a graft composed of endothelium and stroma is inserted into the anterior chamber [7]. In 2004, Melles and colleagues and Price and colleagues improved this technique and published a report on Descemet stripping endothelial keratoplasty (DSEK) [8, 9].

The advantage of this technique lies in the development of a method called *descemetorhexis*, in which the patient’s DM is removed. This descemetorhexis technique improves graft adhesion. Moreover, the anterior stroma is removed by use of a microkeratome during graft preparation in Descemet stripping automated endothelial keratoplasty (DSAEK), allowing for the creation of grafts of uniform thickness and clarity [10]. Adhesion of the clean posterior surface of the patient’s cornea to the anterior surface of the graft has resulted in better visual acuity than that obtained with DSEK or PLK. In Japan, a report analyzing the primary diseases and procedures related to corneal transplantation at a single institution over the past 27 years revealed that the percentage of EK increased, to 33.8% [11]. Aphakic BK, pseudophakic BK, or argon laser-induced BK are common causes of BK [2, 12, 13]. The percentage of EK increased to 33.8%, whilst the prevalence of DMEK lagged behind those of Germany and the United States, and DSAEK was mainly used for EK. BK was the primary disease in 34.5% of the patients.

Currently, the postoperative visual acuity achieved by Descemet membrane endothelial keratoplasty (DMEK), in which only the corneal endothelium and DM are transplanted, without the corneal stroma, is excellent [14]. Compared with DSAEK, DMEK requires a longer learning curve, including aspects such as graft attachment, preparation, and graft unfolding, and offers lower rejection and longer survival time. Because of these advantages, DMEK has become the most commonly chosen EK type in recent years, with the number of DMEK procedures increasing [3, 15].The development of corneal endothelial transplantation has led to a decrease in the number of all-layer corneal transplants. According to a German report, in 2012, more DMEK surgeries were performed than DSAEK surgeries; by 2015, more than 50% of corneal transplants were endothelial transplants [3].

### Immune mechanism in endothelial keratoplasty

**Fig. 3:**
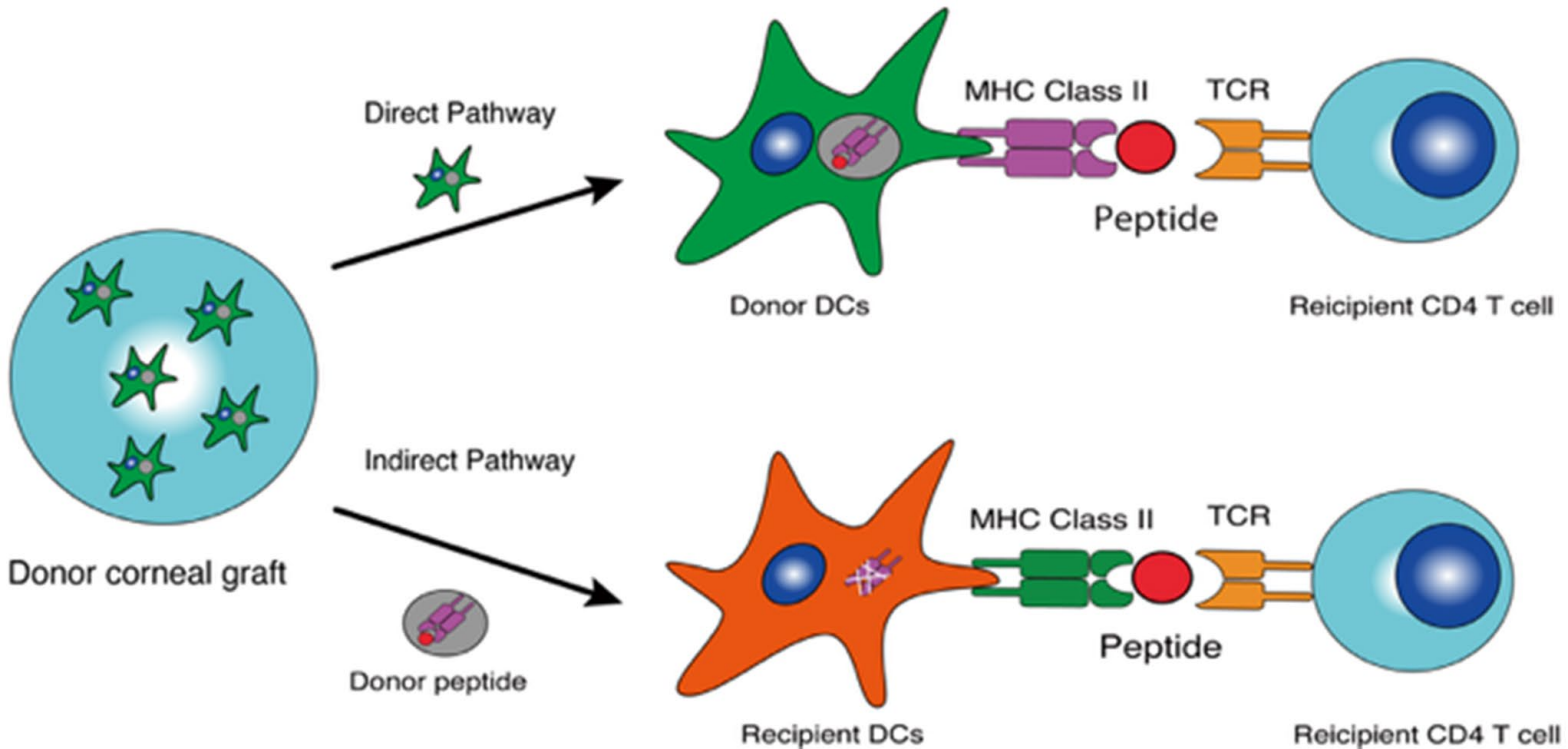
Illustration of mechanism for antigen presentations. In the direct pathway, donor dendritic cells resident in the corneal graft migrate to the cervical lymph node and present to clusters of differentiation 4 (CD4) cells. In the indirect pathway, a peptide with donor-derived major histocompatibility complex (MHC) antigens is recognized by host antigen-presenting cells (APCs) to T cells

The cornea, being a nonvascular and nonlymphoid tissue, has a lower likelihood of presenting antigens when compared with other organs. Therefore, no necessity exists for ethnicity, blood type, or human leukocyte antigen (HLA) matching. The alloantigen recognition mechanism involves direct and indirect pathways (Fig. 3).

In the direct pathway, recipient CD4 and CD8 cells resident in cervical lymph nodes recognize intact major histocompatibility complex (MHC) class I and II alloantigens present on the surface of antigen-presenting cells (APCs). In the indirect pathway, organ-derived peptides (typically MHC antigens) drain into the lymph nodes and are recognized by resident APCs. The indirect pathway is considered the predominant pathway in conventional corneal transplantation, whereas in high-risk settings, such as inflamed or vascularized corneas, both the direct and indirect pathways are involved [16].

Anterior chamber-associated immune deviation (ACAID) is a phenomenon in which the systemic immune response is specifically suppressed against antigens that have entered the anterior chamber [17]. CD4^+^ regulatory T cells are induced by ACAID and have been shown to exert anti-inflammatory effects in autoimmune uveitis and optic neuritis in mouse models [17–19]. Although corneal transplantation has been suggested to be associated with ACAID and has a higher survival rate than other organ transplants, the precise immunologic mechanism is still unclear. However, one case report indicated that splenectomized patients who underwent posterior keratoplasty did not experience graft rejection [20], suggesting that an intact spleen might not be necessary for graft acceptance.

Studies using several mouse models have shown that it is important to prevent recognition of donor tissue or organs as an antigen by the host’s immune system. Replacing allogeneic donor tissue with syngeneic epithelium protects orthotopic allogeneic corneal grafts transplanted into high-risk eyes from sensitization to the recipient, as well as from immune-mediated rejection [21–23]. Reconstitution of BALB/c corneas replaced with an immortalized C3H-CEC cell line in BALB/c mice resulted in no rejection [24].

The allograft rejection rate was further reduced by changing allografts to endothelial transplants. PKP typically has a reported rejection rate of approximately 20%, whereas for EK, the graft survival rate is approximately 7% for DSAEK and 1.5% for DMEK, which is much lower than that for PKP [25, 26].

## Types of keratoplasty, indications, and complications

### PKP

#### Indications

The advantage of PKP is that it can be performed regardless of whether the endothelium or stroma is involved. Therefore, PKP is generally indicated for diseases extending from the corneal stroma to the corneal endothelium. PKP is the treatment of choice in cases of keratoconus, acute hydrops, corneal perforation (infected or noninfected), or graft dysfunction after PKP with stromal opacity.

Overall, PKP has been declining among corneal transplants performed by US eye banks between 2015 and 2020 [15]. PKP is the most common option for repeated surgeries [15]. Corneal thinning due to dilatation or keratoconus is the second-most common cause of PKP. A multicenter study in Germany and China reported a similar downward trend in PKP, with PKP for endothelial diseases being largely replaced by EK [3, 27]. In Australia, corneal graft registries have been analyzed; in particular, PKP for FECD has mostly been replaced with EK [28].

#### Surgery, outcome, and complications

In PKP, the host cornea is removed in a circular shape by use of a vacuum trephine (Fig. 2b). The graft is punched 0.25–0.5 mm larger than the removed cornea and sutured with 10 − 0 nylon, with care taken to avoid induced astigmatism.

A characteristic feature of PKP is its open sky situation during surgery. In the case of cataract surgery, vitrectomy, or EK, the eye is closed, and the shape of the eye and intraocular pressure (IOP) are maintained. In PKP, the eye is in an open sky; thus, increasing the risk of expulsive and choroidal hemorrhage. The operative time should be shortened as much as possible to minimize diversion. A scleral ring is used to maintain the shape of the eye and facilitate surgery. Surgeons must be well trained because of suture-induced astigmatism. Depending on the degree of astigmatism, hard contact lenses may be necessary to achieve good visual acuity [29, 30]. Patients who underwent PKP for BK achieved best-spectacle corrected visual acuity (BSCVA) of 0.73–0.43 at 5 years [31]. In keratoconus cases, visual acuity and rejection rates were better than those in BK cases [32].

The most commonly reported postoperative complications are rejection (approximately 20%), postoperative astigmatism, infectious keratitis (approximately 3.0%), and suture-related infections (approximately 1.0%) [31, 33, 34]. Previous studies have reported that up to one-fifth of patients are left with astigmatism of 5 diopters (D) or more [6, 29, 35, 36]. In cases of keratoconus, visual acuity 1-year post-PKP was 20/40 in 91% of instances [37].

### EK (DSAEK, DMEK)

#### DSAEK

##### Indications

The indications for surgery are similar for both DSAEK and DMEK, being widely performed in cases of endothelial disease without severe scar formation in the stroma. The presence or absence of fibrotic changes in the corneal stroma has been suggested as one of the most important factors affecting visual acuity after DSAEK [38, 39]. Indications include FECD, pseudophakic BK, BK after argon laser iridotomy, failed PKP, post glaucoma surgery, and graft dysfunction after corneal endothelial surgery. Other indications include corneal edema caused by forceps delivery, iridocorneal endothelial (ICE) syndrome, and congenital hereditary endothelial dystrophy (CHED) [26]. The advantage of DSAEK over DMEK is that DSAEK grafts are more rigid than DMEK grafts because they contain stroma. Therefore, DSAEK is selected for patients with corneal opacity and poor visibility of the graft in the anterior chamber because it carries a lower risk of the graft being inserted upside down.

##### Surgery

Donor: The DSAEK graft is prepared using a microkeratome with a 300-, 350-, or 400-µm section. After removal of the anterior part of the cornea, the desired thickness of the posterior donor lenticle is 100 to 150 μm. The size of the trephinations is approximately 8.0 mm.

Patient: The anterior chamber is replaced with air and the DM is removed using a reverse Sinskey hook. The graft is gently inserted into the anterior chamber through the scleral or corneal tunnel by use of forceps (taco-folding method) or an insertion device (Busin glide and forceps, EndoGlide, or NS Endo-Inserter) [25, 40–44]. Air is carefully injected underneath the inserted graft to allow for unfolding and attachment. The tunnel is closed with two or three 10 − 0 nylon sutures (Fig. 2c). The reader should note that the DSEAK procedure described in this section is based on the authors’ preferences and that many other variations exist. There is also a variant called non-Descemet stripping and automated endothelial keratoplasty (nDSAEK), in which the graft is attached without descemetorhexis [45]. Some clinical studies have reported no significant differences in postoperative visual acuity between nDSAEK and DSAEK. The nDSAEK procedure is a simple operation without a DM step, thus representing a useful option for performing EK [39, 45–48].

##### Outcomes and complications

One systematic review reported that the mean visual acuity (BSCVA) after surgery was 0.293 (logarithm of the minimum angle of resolution [logMAR]) [49]. Most other reports that have been published regarding the long-term postoperative observation period have reported a value of approximately 0.40 (logMAR) for BSCVA [50–52]. The refractive index changes by approximately + 1.00 D owing to the effect of changes in graft thickness [26, 53–55]. The 5-year endothelial cell loss rate is approximately 50% [40, 56–58]. The loss rate of ECD was greater than that of PKP.

Common complications of EK (graft preparation and surgical procedures) must be discussed separately. The keratome may not properly resect the cornea during graft preparation. In other words, the donor graft may not be resected with a uniform thickness or tears in the middle of the graft. Therefore, carefully positioning of the keratome blade at the end to ensure proper graft preparation is crucial.

In some cases, the descemetorhexis is inadequate. If the DM is adherent to the stroma owing to trauma, it may not detach with a reverse Sinskey hook. If the DM is rubbed too hard, the graft may not adhere to the tissue; thus, the DM is removed with forceps, or a portion of the DM is cut with a shear blade.

One study conducted a 5-year long-term follow-up of the postoperative results of both DSAEK and nDSAEK and reported no statistical difference in visual acuity, SE corneal endothelial cell density, reduction rate, or other relevant parameters between the 2 patient groups, leading to the conclusion that DSAEK produced favorable results [46].

The primary graft failure rate is 0.62–2.7% [28, 59]. Air or gas injected into the anterior chamber for graft attachment migrates to the back of the iris and causes loss of the anterior chamber; rebubbling is required in 0–20% of cases, which is lower than for DMEK [40, 60, 61]. Graft detachment is reported to be 0.7–0.9% and less than 1% [62, 63]. Another meta-analysis study reported a detachment or dislocation rate of 0–13.3%, indicating differing results across studies [25, 49, 64, 65]. The rejection rate is lower than that of PKP and higher than that of DMEK, although primary and secondary graft failures occur [26, 49].

Thus, the postoperative results of DSAEK are between those of PKP and DMEK, and DSAEK is sometimes recommended in EK where DMEK is not feasible [66, 67], making it an important technique despite its decreasing use.

##### New DSAEK technique

Reports from various countries have shown that DMEK cases are increasing in EK [3, 27]. The advantage of DSAEK is that it is rigid because of the tissue containing stroma, resulting in a reduced risk of backside insertion when compared with DMEK. The DSAEK technique has evolved to provide good and stable postoperative vision.

Busin and colleagues reported an ultrathin DSAEK (UT-DSAEK) with the thinnest possible stroma because the endothelium retains functions important for maintaining corneal transparency [68]. The UT-DSAEK is a 100-µm graft, whereas the conventional graft is approximately 200 μm, and it shortens the time of visual improvement and improves the best visual acuity when compared with DSAEK. The hyperopic shift due to increased corneal thickness can also be reduced to approximately 0.78 D [68].

The standard procedure is to prepare the donor cornea using a microkeratome; however, femtosecond laser ablation has also been reported [69]. The advantage of using a femtosecond laser is that the graft can be fabricated with any thickness. Several studies have reported poorer visual outcomes after femtosecond laser-assisted DSEK than after microkeratome-assisted DSAEK [69]. Recent studies have shown that a new generation of femtosecond lasers, using lower pulse energies and tighter spots, can enable smoother stromal interfaces [70, 71].

The graft insertion technique has also been developed. Although EK has many advantages for endothelial dysfunction, endothelial cell (EC) loss is greater than that in PKP [42, 72–74]. Mechanical injury during surgery is a major cause of postoperative EC loss. An NS Endo-Inserter (Hoya) is a useful option for reducing the traction stress on the endothelium [42]. Soma and colleagues reported that the device differs from the pull-through technique in that the graft is rolled and inserted into the anterior chamber with a balanced salt solution (BSS) flow [42, 75]. The device has a platform to attach the graft to the end of the polypropylene body and a movable polypropylene cartridge attached to a conduit with a silicone rubber valve. Before use, a 2.5-mL syringe filled with BSS is connected to the tip of the inserter body. Moreover, this device needs a 4.6-mm scleral wound to insert the graft, which is smaller than that required for DSAEK. The major advantage of this device is the lack of mechanical traction stress compared to the Busin Glide, which inserts a 1-step graft. However, to date only the NS endo-inserter has been available in Japan. Soma and colleagues reported predominantly less ECD loss at the 6-month follow-up after DSAEK [42]. Combining this device with a frown incision represents another useful technique for reducing the size of the incision [76].

#### DMEK

##### Indication

Compared with DSAEK, DMEK has the advantages of shorter postoperative recovery time, lower rejection rate, and smaller postoperative refractive error change [25, 77, 78]. The indications are the same as those for DSAEK; however, DMEK is considered more cost-effective than DSAEK and is preferred [79].

However, in patients with complex situations such as large iris defects due to large iridectomy (postglaucoma surgery), the existence of a tube shunt for complex glaucoma, a remarkably deep anterior chamber due to high myopia, or postvitrectomy and post-PKP, DMEK is challenging to complete in terms of unfolding or risk of the graft dropping into the vitreous cavity. Thus, the DSAEK is sometimes recommended for difficult situations.

Patients undergoing iridectomy face a risk of the graft falling into the vitreous cavity. Nine eyes that underwent DMEK for aphakia were analyzed, and only 1 eye was reported to be alive at the last follow-up [80]. The authors concluded that DMEK is not recommended for patients with iris defects or aphakia [80]. Once the DMEK graft is dropped, a vitrectomy is required to pull it up, and rescue becomes difficult once it becomes entangled in the vitreous. Second, postglaucoma surgery and/or tube shunt insertion are difficult to perform because the IOP does not increase, potentially causing graft detachment. This is a common difficulty encountered in EK.

For successful unfolding of the DMEK graft, a shallow anterior chamber is required, with the graft positioned between the iris and the cornea. In cases of a significantly deep or vitrectomized anterior chamber, achieving a shallow chamber becomes challenging, making graft deployment difficult. According to a previous report on clinical outcomes of DMEK, 65% of intraoperative complications occurred within the 20 cases that involved vitrectomy, and another 10% of patients showed iatrogenic primary graft failure [66].

In DMEK after PKP, a gap exists between the PKP graft and the host corneal edge of the posterior corneal surface, and the irregularity of the posterior corneal surface also increases the risk of graft detachment [81, 82]. Graft detachment can occur in up to 50% of cases, and it happens with considerable ease [81].

##### Surgery

DMEK grafts were prepared by the surgeon by use of a peeling technique with stained 0.1% Brilliant Blue G or Trypan blue dye [14, 83, 84]. Alternatively, prestripped donor tissues or preloaded grafts could be used [85]. The peeled DMEK graft was loaded into an IOL inserter or glass pipette [86, 87]. The donor tissue was punched for the estimated size (approximately 8.0 mm). After removal of the host’s DM (descemetorhexis) under air infusion, a DMEK graft was implanted into the anterior chamber through a 2.2–2.8 mm corneoscleral tunnel. Subsequently, it was unfolded and fixed by use of 20% SF6 gas. Peripheral iridectomy (PI) was performed at the 6-o’clock position by use of a 25-gauge vitreous cutter. PI can be performed preoperatively by use of an argon laser. Notably, the above-mentioned techniques are similar to DSAEK and vary between surgeons.

##### Outcome and complications

**Fig. 4:**
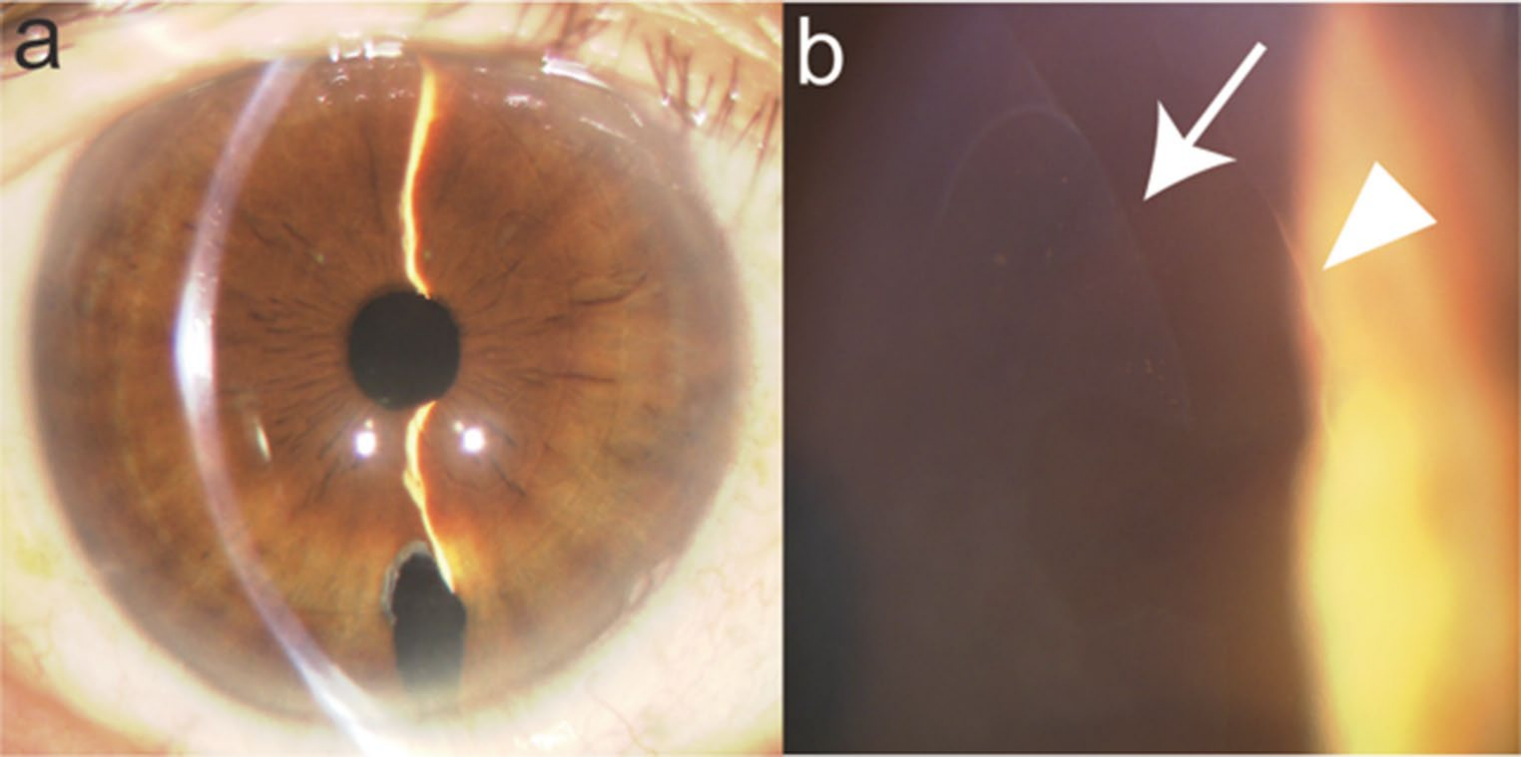
Anterior segment photograph after Descemet membrane endothelial keratoplasty. **a** The transparency of the cornea is remarkably high. **b** The border between the host endothelium (arrowhead) and the graft (arrow) is visible under high magnification

Postoperative visual acuity was better with DMEK than with PKP or DSAEK [25, 49]. Owing to the simple replacement of the thin DM, post-DMEK eyes looked like eyes after cataract surgery (Fig. 4, left). Careful inspection of the corneal periphery revealed a boundary between the removed DM and graft (Figs. 2d and 4, right). Postoperative visual acuity was between 0.08 and 0.3 (logMAR) [49, 78, 88].

Owing to the difficulty of unfolding and confirming the correct orientation, the learning curve until technical stabilization was steeper than that of DSAEK [87–90]. The learning curve for DMEK is severe, requiring experience of approximately 50 cases. With increased DMEK experience, the graft unfolding time decreases, and the decline in ECD lessens [91, 92]. ECD loss rate 5 years after DMEK surgery ranged from − 35% to -80% [31]. However, at 24 months postoperatively, the ECD loss in the DSAEK and DMEK groups seemed to stabilize. Following PKP, ECD continuously decreased [93]. The postoperative graft detachment occurs more frequently with DMEK than with DSAEK [40, 49, 60]. Rebubbling is necessary if the graft becomes detached. Reports exist of cases where the graft is inserted upside-down, although such instances are infrequent. The rate of reverse fixation was approximately 0–18% for DMEK [94, 95]. The primary graft failure was 12.6%, which was higher than that after DSAEK and DSEK (5.7% and 2.7%, respectively) [28].

A meta-analysis comparing DSAEK and DMEK revealed a lower incidence of rejection worldwide for DMEK: the rates were approximately 1.5% for DMEK and 5% for DSAEK [96]. Some reports support or reject lifetime steroid use [96–99]. Follow-ups should be performed with attention paid to the increase in IOP caused by topical steroid eye drops.

However, postoperative graft detachment is more likely to occur with this procedure than with DSAEK [25, 96]. In some cases, when primary PKP grafts fail, DSAEK or DMEK procedures are selected to replace the failed endothelium instead of another PKP being performed. One systematic review reported that patients who underwent DMEK after failed PKP achieved better vision than those who underwent DSAEK [65].

Glaucoma, one of the main complications, was followed until the 36th month, with an incidence of 18.8% for glaucoma and of 12.9% for glaucoma secondary to steroids [100]. The other major complication is postoperative cystoid macular edema (CME) [101, 102], although the detailed mechanism is unknown. Even if EK surgery is completed without complications, the appearance of CME causes a significant loss of vision [103]. The incidence of CME after DSAEK ranges from 7 to 11% [101, 104, 105]. The incidence of CME was 13% in DMEK patients with triple DMEK and DMEK alone, with no difference in the incidence of CME. The presence of iris pigment cells was investigated; however, no association was observed between iris color and CME [106, 107]. We reported a CME incidence of 15.6% in a multicenter study, with the degree of iris damage, air volume, and air reinjection as risk factors [103].

**Fig. 5:**
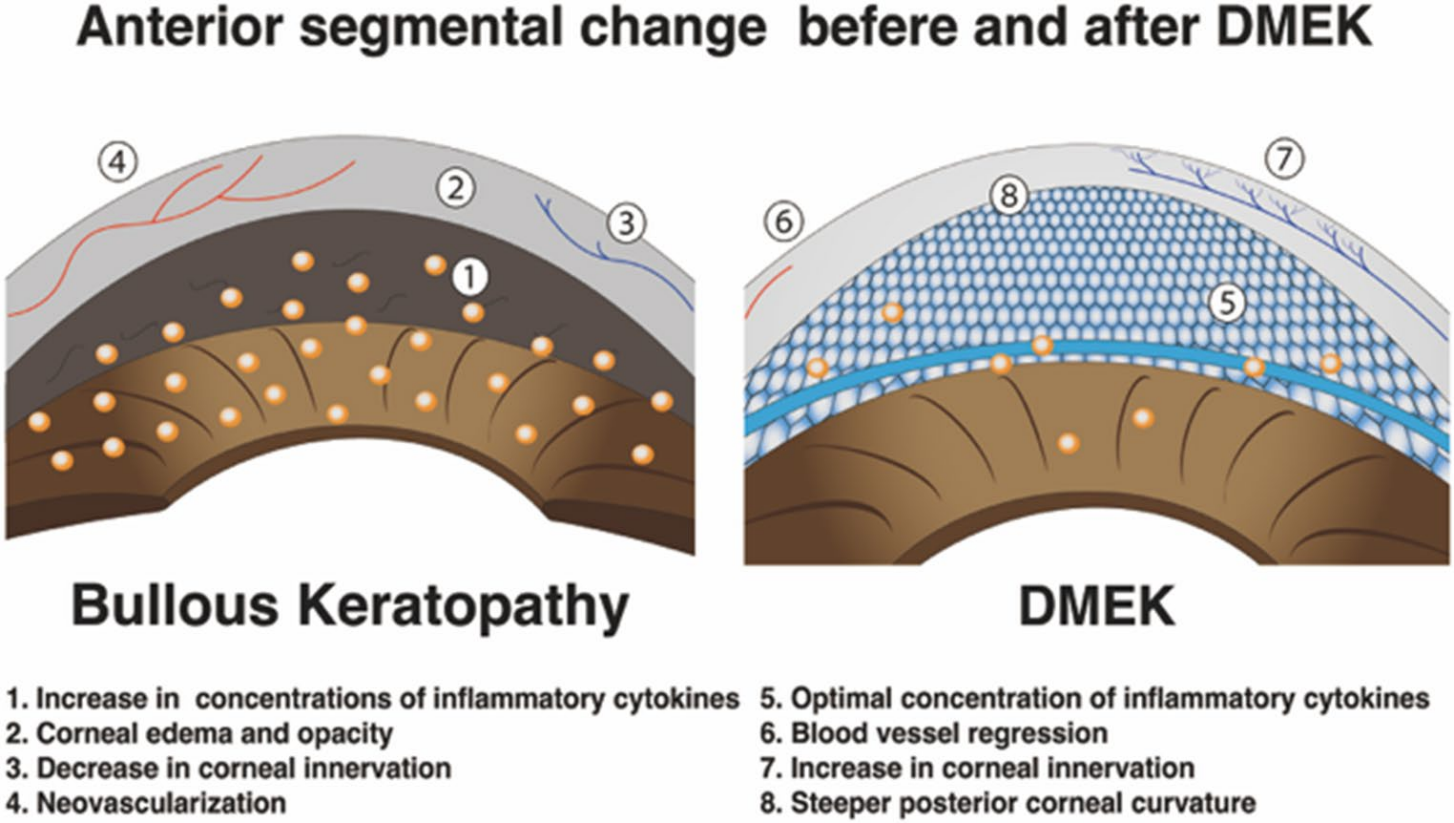
Illustration of the anterior chamber environment before and after DMEK. Before DMEK surgery, the concentration of inflammatory cytokines is high. After DMEK, the anterior chamber environment normalizes owing to the pump mechanism of the corneal endothelium. *DMEK* Descemet membrane endothelial keratoplasty

The severity of iris damage directly correlates with the likelihood of development of posterior iris synechiae [108]. In 1988, Sawa and colleagues reported a method for measuring protein concentration using a laser flare-cell photometer to evaluate flare intensity and the number of cells in the anterior chamber, which is commonly used because of its high reproducibility and noninvasive nature [109, 110]. Noting that inflammation is induced after ocular surgery, a detailed analysis showed changes in cytokine levels after DMEK. Multiplex bead immunoassay revealed that DMEK was associated with a decrease in the number of cytokines in the anterior chamber [111]. Multiplex beads immunoassay results showed that the DMEK group exhibited significantly lower concentrations of several proinflammatory cytokines, such as interleukin-1beta (IL-1β), IL-5, IL-6, IL-10, and IL-8 and granulocyte colony-stimulating factor than those of the BK group. Moreover, the levels of IL-1β and IL-5 were significantly lower in the DMEK group than in the control group. These results indicated that the normal corneal endothelium may play a role in normalizing the environment in the anterior chamber by functioning as a pump (Fig. 5) [111].

Confocal microscopy revealed that the corneal nerve is reduced in EK despite being remarkably small. However, the cornea regenerates relatively early postoperatively and does not become hypersensitive. However, the fact that the corneal nerve density did not return to normal 2 years after surgery suggests that some morphologic changes may have occurred (Fig. 5) [112].

##### New DMEK technique

**Fig. 6:**
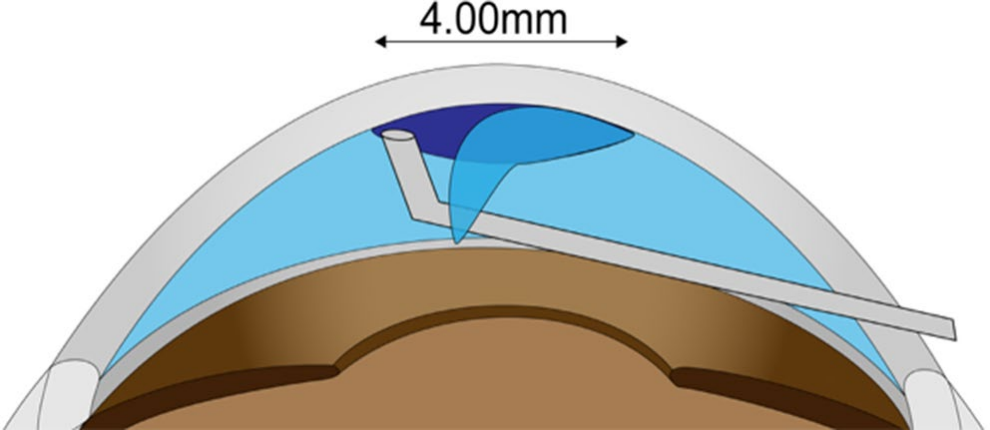
Illustration of Descemet stripping only. The Descemet membrane is stripped using a reverse Sinskey hook of 4.0 mm in diameter

Recently, various techniques have been reported for stabilizing graft unfolding. In general, the standard technique for DMEK is to make the anterior chamber shallow and insert the graft between the iris and cornea, where the graft is unfolded by water flow. In vitrectomized cases, the anterior chamber cannot be made shallower because of the loss of elasticity of the vitreous body. Moreover, if the iris is severely damaged by trauma and/or glaucoma surgery (iridectomy) or if the iris is fixed by mydriasis, the graft might drop into the vitreous cavity. Techniques to prevent graft drops in aphakic eyes have also been reported. A technique named the *safety-net* suture was introduced by Berger and colleagues to prevent graft drop in eyes with aphakia and a large iris defect [113]. In this technique, continuous double-armed 10 − 0 polypropylene sutures were placed in a grid pattern in 3 to 5 horizontal and vertical passes across the full width of the cornea (Fig. 6). The safety-net suture creates a net and forms a temporary barrier, thus reducing the risk of DMEK graft drop with posterior dislocation. The authors performed this technique in 3 patients and concluded that it was a simple and cost-effective solution for preventing graft drop. However, a disadvantage of this technique is the need for secondary pupilloplasty and postoperative iris contact lens wear.

Conventionally, DSAEK is recommended for vitrectomy or severe iris damage. However, owing to its superiority, techniques have been developed to perform DMEK in difficult cases. Our group previously reported the double-bubble technique [114]. This unfolding technique was used for DMEK during vitrectomy. The technique involves injecting 0.02 mL of gas inside the roll of the graft and then inserting additional gas underneath the graft. The graft is unfolded by holding it between the gas, and the unfolding time was 10.1 min.

Furthermore, determining the correct orientation of the graft inserted into the anterior chamber poses a challenge. We also reported a technique for preventing reverse unfolding [115]. BK is a common indication for EK in Japan [116], as are the causative diseases of DMEK. Along with our research team, Kobayashi and colleagues have also reported on various techniques for improving graft visibility using chandelier illumination [117, 118]. Recently, optical coherence tomography (OCT) has been connected to an operating microscope, making it possible to confirm the orientation of graft roles in real-time during surgery [119].

## Descemet stripping only/Descemet membrane without keratoplasty

Recently, with the development of DMEK, even early cases of postoperative FECD have been treated with DMEK when photophobia is severe. In FECD, cystic corneal endothelial cells are observed as guttae, which cause visual loss owing to their diffuse reflections. Because some corneal endothelial cells with normal function are present in patients with FECD, an alternative treatment to transplantation was reported by Kaufman and colleagues in 2018 [120].

Descemet stripping only (DSO) or Descemet membrane without keratoplasty (DWEK) is performed to remove the endothelium of the cornea from the DM in patients with FECD and photophobia. The procedure is performed to improve the symptoms through the migration and expansion of the surrounding normal corneal endothelial cells. The most significant feature of this surgery is the rejection rate of 0%. The risk of secondary glaucoma is greatly reduced without the need for long-term steroid use, which is important after transplantation. The 5-year follow-up results have been positive, and topical use of ripasudil hydrochloride hydrate (Y-27,632) has been reported to promote corneal endothelial cell proliferation [121].

### Indications

The cornea must maintain its function through the migration and expansion of endothelial cells. FECD, with adequate endothelial function, is the most suitable disease for DSO [122]. The inclusion and exclusion criteria used by Moloney and colleagues [121] are summarized as follows:

### Inclusion criteria

1. Grade on Krachmer grading 2–4 with FECD.
2. Clear peripheral endothelial cell count > 1000 cells/mm^2^ on confocal or specular microscopy.
3. Visual acuity reduction of Snellen BSCVA less than 6/6 or caused by guttae rather than corneal edema.
4. Photophobia affecting driving.
5. Age 18 years and over.
6. Visual symptoms due to guttae, not cataracts or corneal stromal edema.

### Exclusion criteria

1. Advanced corneal stromal edema defined as the presence of haze, bullae, or DM folds on slit-lamp biomicroscopy.
2. Peripheral endothelial cell count of < 1000 cells/mm^2^.
3. Presence of secondary corneal pathology such as infective or autoimmune keratitis.
4. History of herpes simplex virus or cytomegalovirus keratitis.
5. Pregnancy.

### Surgery

In a previous study, DSO was performed at 6 mm; however, corneal transparency was not recovered completely [97]. Removing the DM at a 4.00-mm diameter is now widely documented (Fig. 6). If the cornea does not regain function for several months after surgery, an EK is performed. Under topical anesthesia, a 4.00-mm descemetorhexis is conducted in the central area with the aid of viscoelastic material, using either a reverse Sinskey hook or a Fogla DM stripping hook. Care should be taken when peeling to prevent expansion of the peeled area.

### Complications

The primary focus of observation is prolonged corneal edema. If the edema does not improve over several months, EK should be considered before fibrotic changes occur in the stroma [123, 124]. In other instances of DM detachment, air injection might prove effective. Caution should be exercised because intense abrasion of the posterior surface of the cornea may cause scarring in the posterior stroma.

## Future treatment

Corneal transplantation has made great progress by improving postoperative visual acuity and decreasing the incidence of rejection. However, we encounter severe cases that require repeated corneal endothelial transplantation after glaucoma surgery or because of cytomegalovirus-induced corneal endotheliitis. Repeated use of endothelial grafts shortens graft survival. In response to this situation, several alternatives are being developed that represent a significant change from previous concepts.

The first is the injection of cultured corneal endothelium. Until now, a sheet graft containing the corneal endothelium, DM, or stroma has been inserted into the anterior chamber, as described by Kinoshita and colleagues [125]. Patients undergo ablation of diseased corneal endothelial cells, as in the DSO procedure, in which an 8.00-mm area is abraded; corneal endothelial cells are cultured ex vivo, injected, and maintained in the supine position for successful implantation. Although this is a new technique, good results have been reported 5 years postoperatively. Unlike in conventional endothelial transplantation, the patient is placed in the supine position for cell adhesion. Furthermore, the incision is 1.6 mm, which is exceedingly small. One problem is the need for healthy, relatively young human corneal endothelium for culture. The second method is corneal endothelial transplantation using embryonic stem (ES)/induced pluripotent stem (iPS) cells, as reported by Hatou and colleagues [126, 127]. Unlike the report by Kinoshita and colleagues, endothelium differentiated and cultured from iPS cells has the advantage of reduced susceptibility to rejection and does not require a donor [126]. Currently, successful induction of corneal endothelial cell differentiation from iPS cells is limited to experimental animal models, and clinical application is anticipated. The potential clinical application carries numerous benefits, such as shortening of the waiting period before transplantation and reduction of rejection rates.

Various methods have been explored to address recurrent rejections with artificial interventions. Recently, clinical use of artificial corneal endothelium (EndoArt; EyeYon Medical) has gained traction [128]. These grafts have a dome-shaped structure, 50-µm thickness, and diameter of 6.0 mm. Artificial corneal endothelium, composed of a flexible, hydrophilic-acrylic material, has replaced corneal endothelial disease. Evaporation of the epithelium and reduced aqueous humor inflow into the central stroma ultimately reduce edema and restore corneal homeostasis. The surgical technique closely resembles that of DMEK, with a notable emphasis on ensuring secure attachment to the posterior corneal surface owing to its nonbiologic nature. The authors report that in 2 patients with a preoperative corneal thickness of over 700 μm, the corneal thickness could be reduced and kept to approximately 500 μm after 1 year of postoperative observation. The possibility of substituting an artificial material would be beneficial for these patients because ECD loss progresses owing to the deterioration of the anterior chamber environment, such as elevated inflammatory cytokines [111]. As the number of reported cases is limited, further studies are required.

## Discussion

This review provides a comprehensive overview of the history, indications, techniques, and prospects of corneal endothelial transplantation.

Although endothelial transplantation is a relatively new technique, several reports have already been published comparing DSAEK and DMEK, with DMEK typically reported as better in terms of rejection rate and postoperative visual acuity [25, 52, 60, 129]. Both also have good postoperative outcomes; however, the accumulation of surgical data underscores the existence of difficult cases for which careful consideration is essential when deciding whether to perform endothelial transplantation, particularly when considering DMEK. The general consensus is that patients with unstable anterior chambers require careful consideration and that other surgical options should also be considered. Recently, techniques have made it possible to perform endothelial transplantation in difficult cases, and the range of indications for endothelial transplantation has expanded. The number of DMEK cases continues to increase worldwide.

More than 20 years have passed since the development of the DMEK technique, and its postoperative course is stable. Moreover, an assessment encompassed both general and intricate aspects, including evaluations of grafts, visual function, the frequency of glaucoma, and changes in corneal shape. Glaucoma represents one of the major complications. In an analysis of 90 DMEK cases, elevated IOP and pseudoexfoliation syndrome were reported as a risk factor in 21% of the patients [130]. Corneal transparency showed a remarkable improvement, and the visual outcome was favorable. Nevertheless, the assessment of corneal higher-order aberrations indicated a deterioration, notably in the posterior cornea, where the central region exhibited anterior protrusion (Fig. 5) [131]. This observation suggests that the cornea goes through a process of normalization and structural alterations [131–133].

Furthermore, the success of DMEK in difficult cases—such as those with iridocorneal endothelial syndrome following PKP and tube shunts after glaucoma surgery—is key to its future as a preferred treatment option [100, 134, 135]. Because of good surgical results, the development of this method is now moving toward simplification of the procedure. In conjunction with modern technology, attempts have been made to predict graft detachment after DMEK using artificial intelligence [128, 136].

The use of new technologies could solve the shortage of donor organs. The mechanism of the artificial endothelium (EndoArt) involves blocking contact with the aqueous humor. The strengths of this surgery include the lack of long-term steroid use to prevent rejection [128].

Cell injection therapy using corneal endothelial cells derived from iPS or cultured cells supplies donor cells and is reproducible, eliminating the need for complex DMEK unfolding techniques. However, the requirements of regenerative medicine should be considered in terms of the regulations and costs.

The field of endothelial corneal transplantation has great potential for future advances, with continued growth expected to improve visual acuity and reduce the need for repeated transplantations.
